# PhyloSort: a user-friendly phylogenetic sorting tool and its application to estimating the cyanobacterial contribution to the nuclear genome of *Chlamydomonas*

**DOI:** 10.1186/1471-2148-8-6

**Published:** 2008-01-15

**Authors:** Ahmed Moustafa, Debashish Bhattacharya

**Affiliations:** 1 University of Iowa, Interdisciplinary Program in Genetics, 456 Biology Building, Iowa City, Iowa 52242, USA; 2 University of Iowa, Department of Biological Sciences and the Roy J. Carver Center for Comparative Genomics, 446 Biology Building, Iowa City, Iowa 52242, USA

## Abstract

**Background:**

Phylogenomic pipelines generate a large collection of phylogenetic trees that require manual inspection to answer questions about gene or genome evolution. A notable application of phylogenomics is to photosynthetic organelle (plastid) endosymbiosis. In the case of primary endosymbiosis, a heterotrophic protist engulfed a cyanobacterium, giving rise to the first photosynthetic eukaryote. Plastid establishment precipitated extensive gene transfer from the endosymbiont to the nuclear genome of the 'host'. Estimating the magnitude of this endosymbiotic gene transfer (EGT) and determining the functions of the prokaryotic genes remain controversial issues. We used phylogenomics to study EGT in the model green alga *Chlamydomonas reinhardtii*. To facilitate this procedure, we developed PhyloSort to rapidly search large collection of trees for monophyletic relationships. Here we present PhyloSort and its application to estimating EGT in *Chlamydomonas*.

**Results:**

PhyloSort is an open-source tool to sort phylogenetic trees by searching for user specified subtrees that contain a monophyletic group of interest defined by operational taxonomic units in a phylogenomic context. Using PhyloSort, we identified 897 *Chlamydomonas *genes of putative cyanobacterial origin, of which 531 had bootstrap support values ≥ 50% for the grouping of the algal and cyanobacterial homologs.

**Conclusion:**

PhyloSort can be applied to quantify the number of genes that support different evolutionary hypotheses such as a taxonomic classification or endosymbiotic or horizontal gene transfer events. In our application, we demonstrate that cyanobacteria account for 3.5–6% of the protein-coding genes in the nuclear genome of *Chlamydomonas*.

## Background

### Phylogenomics

Recent advances in sequencing technologies and reductions in the cost of sequencing have fostered an unprecedented explosion of complete genome and expressed sequence tag (EST) data and facilitated a revolution in the field of comparative genomics. The availability of this massive amount of genome data has been both a boon and a major challenge for biologists. Phylogenomics offers an useful avenue for dealing with genome data, allowing researchers to investigate gene phylogeny in a genome-wide context [1]. This approach can be used to annotate genes of unknown function in newly sequenced genomes or to identify phylogenetic markers to infer an accurate genealogy of life, or to understand the role of endosymbiotic and horizontal gene transfer in eukaryotic evolution [2]. Existing phylogenomic pipelines (e.g., PhyloGenie [3] and PhyloGena [4]) generate a large collection, often hundreds or thousands, of phylogenetic trees that require manual inspection to observe general patterns of genome evolution or to address specific hypotheses about gene phylogeny.

### Endosymbiosis

The engulfment of a free-living photosynthetic cyanobacterium by a heterotrophic protist (primary endosymbiosis) introduced photosynthesis into the eukaryotic domain. The primary endosymbiosis occurred about 1.6 billion years ago (BYA) [5] and was a turning point for evolution of life on our planet allowing the later development of multicellular plants and animals. The first algae diversified over time into the three primary photosynthetic lineages, the green (later including land plants), red, and glaucophyte algae. These taxa are united in the eukaryotic supergroup Plantae. Some time after establishment of the endosymbiotic relationship there was selective pressure to reduce the endosymbiont genome by outright gene loss or transfer of genes to the host nuclear genome. This latter process is termed endosymbiotic gene transfer (EGT). Determining the extent of 'primary' EGT in Plantae and whether only genes involved in plastid function were retained or were augmented by many other genes of non-organellar function remain controversial issues. Previously, using the flowering plant *Arabidopsis*, it was estimated that the cyanobacterial endosymbiont contributed 18% of the total set of nuclear genes in this species [6]. In another study, EGT was shown to have contributed only about 4% of the nuclear genes in *Arabidopsis*, and 12% to the reduced nuclear genome of the extremophilic red alga *Cyanidioschyzon merolae *[7]. In a more recent study using the free-living glaucophyte alga *Cyanophora paradoxa*, we estimated that about 4% of the nuclear genome in this taxon was of cyanobacterial provenance. However this latter study was based on incomplete EST data and is a provisional result. There exists therefore a need to apply modern methods to analyze the complete genome sequence of a mesophilic, free-living, unicellular alga to generate a robust estimate of primary EGT in a relatively 'simple' ancestor of land plants [2].

Furthermore, once the Plantae split into its constituent lineages, a red (and likely also a green) alga was captured by the ancestor of the chromalveolate protists via secondary endosymbiosis [8]. This process necessitated the movement of genes involved in plastid function from the nucleus of the algal endosymbiont to that of the chromalveolate host through 'secondary' EGT. The chromalveolate supergroup includes a broad swath of protist diversity including both photosynthetic (stramenopile algae, haptophytes, dinoflagellates) and plastid-lacking (oomycetes, ciliates, telonemids) lineages. Presumably the latter group have lost their plastid secondarily. Phylogenomic analysis of nuclear genes in these algae is expected therefore to return trees which show gene origin in Plantae from a cyanobacterial ancestor, followed by their transfer to chromalveolates via a red or green algal secondary endosymbiosis [9] and [10].

Given the need to better understand EGT in algae using a phylogenomic approach, we developed PhyloSort to analyze topologies in a high-throughput fashion. This opensource Java tool inspects the topology of phylogenetic trees to address the most frequently asked question in the field: does a specific gene support the monophyly of certain operational taxonomic units (OTUs; e.g., cyanobacteria and Plantae)? Here, we provide an overview of PhyloSort and its application to the complete set of predicted proteins in the green alga *Chlamydomonas reinhardtii *to estimate the contribution through EGT of cyanobacteria to the nuclear genome of a green alga.

## Implementation

**Figure 1:**
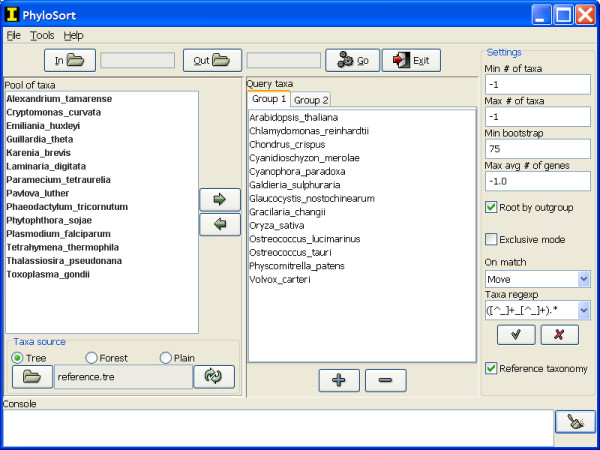
Screenshot of the main window of PhyloSort (Windows XP).

PhyloSort can be used via a graphical user interface (GUI; Figure 1) and a text mode command line interface. Input phylogenetic trees are read and parsed from a source folder, where trees are stored as one tree per file in Newick format, which is supported and produced by many phylogenetic inference tools such as PHYLIP [11], PAUP* [12], PhyML [13] and RAxML [14]. Of the input trees, those that satisfy the search criteria are copied or moved to an output folder.

**Figure 2:**
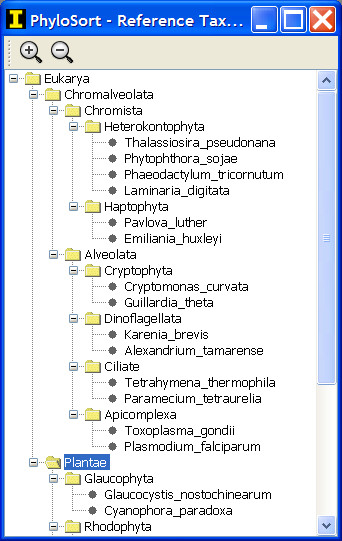
Screenshot of the Reference Taxonomy tree in PhyloSort (Windows XP) that can be used to select the groups of taxa.

To begin the analysis, the hypothesized monophyletic taxa are selected from a pool of taxa. This pool of taxa can be loaded as a simple list from a plain text file. Alternatively, a tree can be loaded that acts as a taxonomy reference for organizing the taxa in a phylogenetic format (Figure 2). Finally, if no list is loaded or no reference tree exists, the program will unite all taxa in all trees into a single non-redundant list of taxa.

In a typical phylogenomic analysis, homologs within and among different genomes give rise to multiple sequence alignments (using, for example, MUSCLE [15] or ClustalW [16]) that do not all necessarily share among them the same set of taxa. Therefore, the number of taxa that represent members of any monophyletic group (e.g., cyanobacteria and Plantae) may (and likely will) vary from tree to tree. This can be explained, for example, by lineage-specific gene duplications or gene losses. For this reason, in PhyloSort, the hypothesized taxa are arranged in groups such that for any number of groups to be monophyletic each group has to be represented by at least one of its constituent taxa. In addition, because the names of the sequences used in reconstructing the trees would have different formats from one project or research group to another, "regular expressions" [17] are used to extract taxa (or species names) from the sequence names.

In addition to the topological constraint of monophyly, the PhyloSort search can be adjusted by setting a minimum bootstrap support value associated with the monophyletic clades. This allows the identification of trees with significantly supported (therefore, more reliable) monophyletic relationships. Minimum and maximum number of taxa in a trees and average number of genes per taxon can also be chosen to sample differing levels of gene family complexity.

PhyloSort offers two search modes, exclusive and inclusive. In the exclusive mode, the taxa that exist in the tree and belong to the hypothesized monophyletic taxa have to be located in a single monophyletic clade. In the inclusive mode, any group of taxa forming a clade that matches the search criteria qualifies the trees regardless of whether other taxa belong to the hypothesized monophyletic taxa exist elsewhere in the tree. Prior to the monophyly search, each tree is searched for an outgroup and then rerooted on that taxon.

A genome-wide analysis generally produces a significant number of trees that share multiple genes due to multiple gene copies and gene families. Accordingly, to provide an estimate of the number of unique gene families, PhyloSort has a simple clustering functionality that can be used to merge trees by identifying overlapping genes among trees and placing the trees into 'tree clusters' representing gene families. A minimum number of overlapping genes can be set for merging trees into clusters.

Through the GUI interface, a taxonomy reference tree (Figure 2) can be used to hierarchically collect taxa and simplify the assignment of taxa into groups, and phylogenetic trees can be visually inspected using ATV [18]. In addition to the GUI and command line interfaces, PhyloSort provides a set of reusable and extendable application programming interfaces (APIs) that can be incorporated into other applications that may utilize the monophyly search or other utility components such as Newick format parsing and basic phylogenetic tree manipulation.

To determine whether a set of taxa is monophyletic, there are two main steps. First, the lowest common ancestor (LCA) is located. Second, the subtree rooted by the LCA is verified to not contain outgroups. The following subsections summarize the approaches that have been implemented to determine whether a tree contains a clade that matches the monophyletic criterion and any additional constraints in both search modes.

### LCA Identification

**Figure 3:**
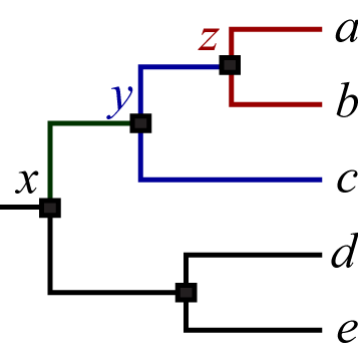
**A graphical cladogram representation of the tree (((*a*, *b*), *c*), (*d*, *e*))**. In the tree, *x *is root, *a*, *b*, *c*, *d *and e are terminals (leaves), and *y *and *z *are internals. To determine whether *a*, *b *and *c *are monophyletic, the following steps are performed: I. The paths from *x *to *a*, *b*, and *c *are (*x *→ *y *→ *z *→ *a*), (*x *→ y → *z *→ *b*), and (*x *→ *y *→ *c*) respectively. II. The longest shared segment among the three paths is (*x *→ *y*). III. The LCA of *a*, *b*, and *c *is *y*. IV. The subtree rooted by *y *contains only *a*, *b*, and *c*. V. *a*, *b*, and *c *are monophyletic in the clade rooted by *y*.

To locate the LCA for a set of leaves (taxa), the path from the root to each leaf in the set is determined. Next, all paths are compared to find the longest shared segment (i.e., number of shared consecutive nodes). Then, the LCA is the furthest node from the root on the longest shared segment (Figure 3).

### Exclusive search

Under the exclusive mode, all taxa in the tree that belong to the set of taxa being examined for monophyly are located and the LCA for the entire set is determined and the clade rooted by the LCA is tested for monophyly.

### Inclusive search

Under the inclusive mode, each leaf that belongs to the groups of taxa being examined for monophyly is used as a starting point to traverse the tree in a tip to root direction. Each internal node is examined as described above in the exclusive search to test whether it contains a qualifying monophyletic clade. If the clade rooted by the internal node has a taxon that does not belong to the hypothesized monophyletic taxa, the clade is rejected, all nodes belong to the clade are marked not to be revisited, and the next leaf is examined. If a clade is not rejected but it does not satisfy the monophyly constraint or any additional filtering criterion, the parent of the clade is located and the clade rooted by the parent is examined. Otherwise, if the clade satisfies all criteria, the search stops returning the tree as a matching tree. This search strategy was specifically designed to identify trees that contain paralogs, a subset of which may satisfy the monophyly criterion and therefore should be considered.

## Results

As an example of the application of PhyloSort to address biological questions, we quantified primary (cyanobacterial) EGT in the green alga *Chlamydomonas reinhardtii*. Of the 15,143 predicted *Chlamydomonas *nuclear-encoded proteins, we found 4,631 that had cyanobacterial homologs with a BLAST e-value < 1e-5 (i.e., ~30% of the total number of genes) for which 4,129 trees were inferred. With no restriction on the bootstrap support value (i.e., bootstrap support value ≥ 0%), there were 897 (~6% of the genes) trees that showed monophyly of cyanobacteria and Plantae (and in many cases, chromalveolates). By enforcing the minimum bootstrap support values at ≥ 50% or ≥ 75%, we found 531 (~3.5% of the genes) and 406 trees, respectively, that satisfied the monophyly constraint. Clustering the 50% bootstrap category resulted into at least 267 unique gene families. Based on the gene ontology analysis (see Methods), at least 44% of these predicted cyanobacterial genes were identified as encoding plastid-targeted proteins and at least 47% were involved in metabolic processes.

## Discussion

**Figure 4:**
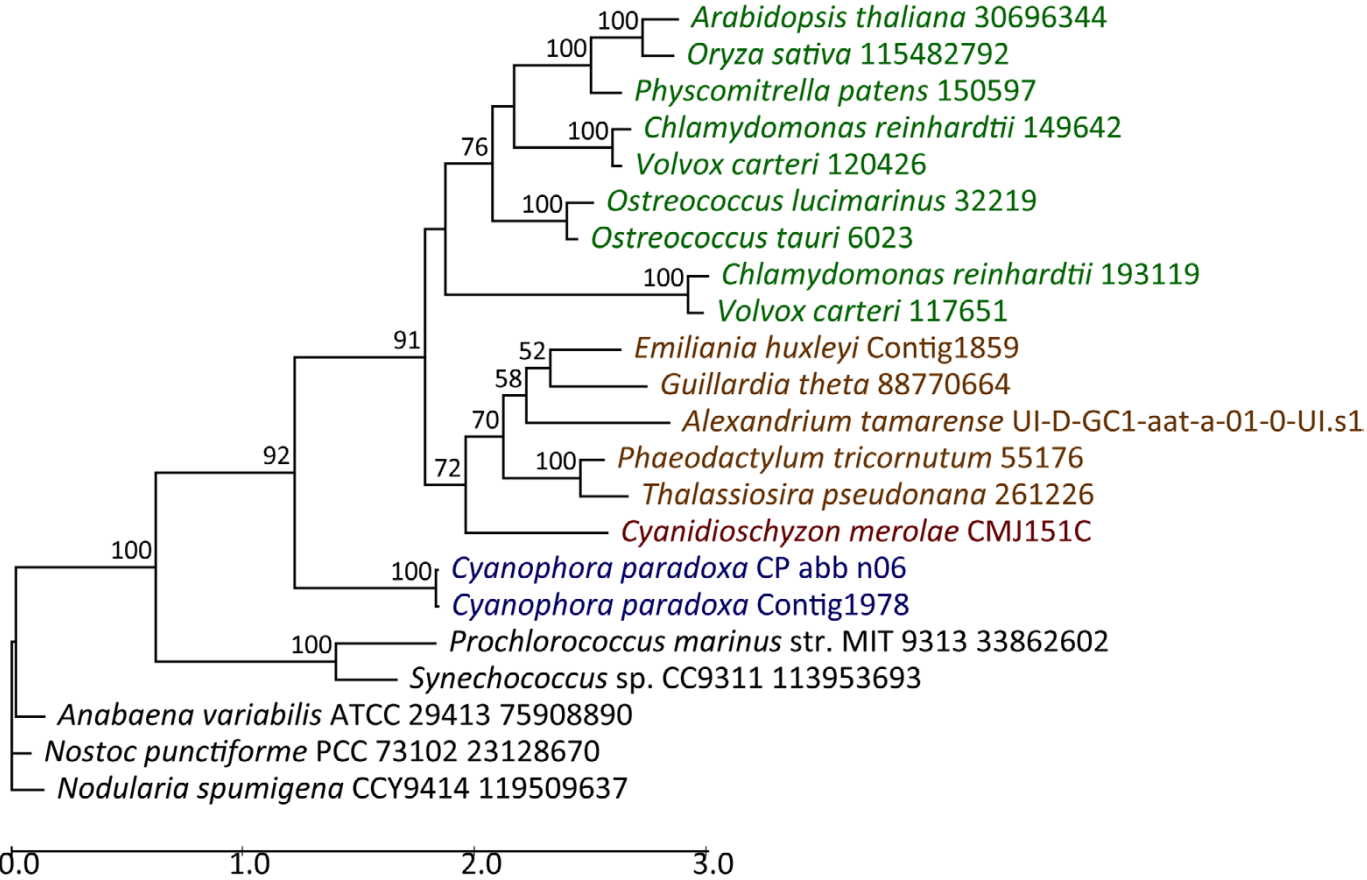
**Phylogenetic tree of thylakoid lumenal 17.4 kDa protein**. This is a maximum likelihood tree inferred using RAxML-VI-HPC, v2.2.1 with the JTT evolutionary model and 100 bootstrap replicates 14 (bootstrap values < 50% are omitted from the phylogeny). We used a random starting tree (one round of taxon addition) and the rapid hill-climbing algorithm (i.e., option -f d in RAxML). The tree was drawn using Drawtree 27. Lineages are color-coded as follows: green → green algae and land plants, red → red algae, brown → chromalveolates, blue → glaucophytes, and black → cyanobacteria.

Of the trees that supported the monophyly of cyanobacteria and photosynthetic eukaryotes (represented by Plantae and chromalveolates), we present here as an example the phylogenetic tree of the thylakoid lumenal 17.4 kDa protein (Figure 4). This plastid-targeted protein belongs to the "pentapeptide repeat" family of proteins of which the function has not yet been characterized in Plantae. However, it has been shown that the pentapeptide repeats are required for a proper localization of heterocyst glycolipids in cyanobacteria [19].

The tree demonstrates the phylogeny predicted for plastid primary and secondary endosymbiosis [2]. The nuclear-encoded Plantae proteins are of cyanobacterial origin (primary EGT), whereas the chromalveolate sequences are rooted within red algae (secondary EGT; 72% bootstrap support value). Here the Plantae are represented by its three member lineages, the glaucophyte *Cyanophora*, the red alga *Cyanidioschyzon*, the green algae *Ostreococcus*, *Chlamydomonas*, *Volvox *and *Physcomitrella*, and the plants *Arabidopsis *and *Oryza*, whereas the chromalveolates are represented by the diatoms *Phaeodactylum *and *Thalassiosira*, the haptophyte *Emiliania*, the cryptophyte *Guillardia*, and the dinoflagellate *Alexandrium*). Previous analyses suggest that the chromalveolate red algal secondary endosymbiosis occurred 'soon' (ca. 1.3 BYA [5]) after the cyanobacterial capture. Many other *Chlamydomonas *proteins display the same topology as shown in Figure 4 and are available as individual trees in [Additional file 1].

Specifically, of the 531 genes that belong to the 50% bootstrap category, about 51% of the genes were shared among red and green algae and about 12% were shared among glaucophytes, red and green algae. The unevenness of the distribution of cyanobacterial genes among the Plantae lineages can be explained by limited EST data that are available from glaucophytes (the nuclear genome is currently being sequenced to completion [20]), the highly reduced nature of the genome of the red alga *Cyanidioschyzon *(16.5 Mb [21]) which likely precipitated the loss of many target genes, and the sampling bias towards the green lineages due to the use of *Chlamydomonas *as the query for the phylogenomic analysis. In summary, using PhyloSort we were able to rapidly inspect thousands of phylogenetic trees of differing complexities and number of terminal taxa and demonstrate the contribution of cyanobacterial primary EGT to Plantae nuclear genomes.

## Conclusion

PhyloSort is a simple, platform independent and user-friendly tool to inspect the topology and other aspects (e.g., bootstrap support values, complexity) of large sets of phylogenetic trees in a phylogenomic context. PhyloSort can be used to quantify the number of genes (or trees) that support different evolutionary hypotheses such as a taxonomic classification or to quantify endosymbiotic or horizontal gene transfer events. In addition, PhyloSort can be used to search for vertically inherited phylogenetic markers, for example across the eukaryotic tree of life, by testing the monophyly in individual gene trees of members of each of the six putative eukaryotic supergroups [22].

Our analysis of *Chlamydomonas *(and other Plantae) demonstrates that cyanobacteria account for ca. 3.5–6% (897 and 531 genes at bootstrap support values ≥ 0% and 50%, respectively) of the protein-coding genes in the nuclear genome of this taxon and that at least 63% of the transferred cyanobacterial genes are present in the red and green lineages. A previous analysis suggested that about 90% of the gene products resulting from primary EGT are targeted to the plastid [2]. Therefore it appears that whereas primary endosymbiosis strongly favored EGT to the nucleus, selection reduced the impact of cyanobacterial gene incursions primarily to functions that are plastid localized. Furthermore, it is now clear that outright loss was the most common fate for cyanobacterial genes in algae because with ca. 1,000 genes in the nucleus and another 150–200 in the plastid, this accounts for less than 50% (i.e., 1,200/2,500 genes in many extant cyanobacteria) of the ancestral endosymbiont genome.

Finally, we discuss briefly the relevance of our findings to the extent of 'real' primary EGT. Our results are based on detecting phylogenetic signal in anciently diverged sequences using standard computational approaches. Given that phylogenetic signal was most certainly lost from many cyanobacterial genes over the greater than 1 billion years since the endosymbiotic event, it will most certainly be impossible to detect all instances of EGT. Therefore, how significant is our number of 531 cyanobacterial-derived genes in *Chlamydomonas *(at the 50% bootstrap value threshold)? In our opinion, this must clearly be viewed as an underestimate of the actual number. Many cyanobacterial genes may simply no longer be detectable as such due to highly diverged sequences. A more accurate estimate may come from increased taxon sampling (e.g., including *Cyanophora *and mesophilic red algae with 'normal-sized' genomes) in the phylogenomic analyses to allow these methods to potentially identify a larger proportion of highly diverged homologs among the Plantae. We also need to gain a better understanding of the process of phylogenetic signal decay among this gene set so that extrapolations to the true value are more realistic.

## Availability and requirements

PhyloSort is available as an open-source under The GNU General Public License (GPL) via WWW at http://phylosort.sourceforge.net. PhyloSort is implemented in Java under compliance with compiler version 5.0. It has been successfully tested on machines running the operating systems Sun OS, Red Hat, Windows, and Mac OS.

## Methods

### Screening *Chlamydomonas *genes for candidates of cyanobacterial origin

The 15,143 predicted nuclear-encoded proteins of the green alga *Chlamydomonas reinhardtii *were used to identify genes with a potential cyanobacterial origin. We combined a comprehensive set of cyanobacterial proteins from 43 different species and strains obtained from RefSeq [23], resulting in a total of 121,252 cyanobacterial proteins. We searched for *Chlamydomonas *nuclear homologs in the combined cyanobacterial set using WU-BLAST [24] and found 4,631 *Chlamydomonas *proteins with significant matches (BLAST e-value < 1e-5) in the cyanobacterial data set.

### Phylogenomic analysis

We compiled a set of 1,066,173 amino acid sequences from complete genomes and partial EST libraries obtained from public and local sources including more than 70 organisms, spanning the major groups of living organisms (43 cyanobacteria, 13 Plantae, 16 chromalveolates, 30 bacteria, 3 amoebae, 5 excavates and 10 opisthokonts). We used the PhyloGenie pipeline [3] to execute the phylogenomic analysis on the 4,631-filtered *Chlamydomonas *proteins against the 1,066,173 protein database; the pipeline produced 4,129 trees using neighbour-joining with Poisson distance correction scheme and 100 replicates of a bootstrap analysis.

### Topology search

Using PhyloSort, we inspected the topologies of the inferred trees looking for clades showing the monophyly of cyanobacteria and Plantae (and chromalveolates). We categorized the matching trees based on the bootstrap support value on the monophyletic clade into three categories, trees with bootstrap support values ≥ 0%, 50%, and 75% and clustered the trees in each bootstrap category into unique gene families. The complete list of trees in the 50% bootstrap support category is available in the supplementary material [see Additional file 1] along with the protein phylogenies and annotations.

### Gene Ontology analysis

We annotated and identified the Gene Ontology (GO) terms (biological process, cellular component, and molecular function) [25] for each gene in the 50% bootstrap support category [see Additional file 2]. We wrote a Perl program "blast2go.pl" to retrieve GO terms from a MySQL GO database [26].

## Authors' contributions

DB led the project. AM and DB designed the software and the phylogenomic study. AM implemented the software and conducted the phylogenomic analysis. AM and DB analyzed the data and prepared the manuscript. All authors have read and approved the final version of the manuscript.

## Supplementary Material

Additional file 1 **Genes and Trees**. The list is presented of the 531 *Chlamydomonas *proteins that form a monophyletic clade with cyanobacteria and other photosynthetic eukaryotes. The list includes the inferred phylogenetic tree for each gene and the associated GO annotation. Click here for file

Additional file 2 **Gene Ontology**. The results of the gene ontology (GO) analysis (format: Microsoft Word Document). Click here for file
