# Supplementary material for: PhyloSort: a user-friendly phylogenetic sorting tool and its application to estimating the cyanobacterial contribution to the nuclear genome of Chlamydomonas
Source: BMC Evol Biol. 2008 Jan 15;8:6. doi: 10.1186/1471-2148-8-6 (PMC2254586; doi:10.1186/1471-2148-8-6)
Supplement: Additional file 2 — Gene Ontology. The results of the gene ontology (GO) analysis (format: Microsoft Word Document). [file 1471-2148-8-6-S2.doc]

## Distribution of Gene Ontology terms within the predicted cyanobacterial genes with a bootstrap support ≥ 50%


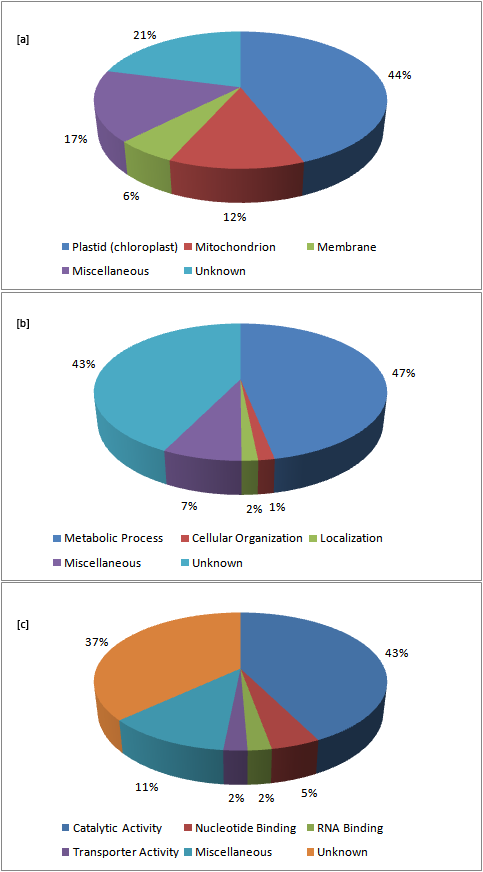


[a] cellular components, [b] biological processes, and [c] molecular functions.
